# Nrf2/ARE Activators Improve Memory in Aged Mice via Maintaining of Mitochondrial Quality Control of Brain and the Modulation of Gut Microbiome

**DOI:** 10.3390/ph14070607

**Published:** 2021-06-23

**Authors:** Irina S. Sadovnikova, Artem P. Gureev, Daria A. Ignatyeva, Maria V. Gryaznova, Ekaterina V. Chernyshova, Ekaterina P. Krutskikh, Anastasia G. Novikova, Vasily N. Popov

**Affiliations:** 1 Department of Genetics, Cytology and Bioengineering, Voronezh State University, 394018 Voronezh, Russia; ira-ivankina@yandex.ru (I.S.S.); bolovintseva2000@gmail.com (D.A.I.); Mariya-vg@mail.ru (M.V.G.); kate.chernyshova166@mail.ru (E.V.C.); kru751@rambler.ru (E.P.K.); novikova.anastasi@yandex.ru (A.G.N.); pvn@bio.vsu.ru (V.N.P.); 2 Laboratory of Metagenomics and Food Biotechnology, Voronezh State University of Engineering Technology, 394036 Voronezh, Russia

**Keywords:** aging, cognitive dysfunction, Nrf2/ARE pathway, mitochondrial biogenesis, mitophagy, antioxidants, resveratrol, dimethyl fumarate, methylene blue, memory, gut–brain axis, gut microbiome

## Abstract

Aging is one of the most serious factors for central nervous dysfunctions, which lead to cognitive impairment. New highly effective drugs are required to slow the development of cognitive dysfunction. This research studied the effect of dimethyl fumarate (DMF), methylene blue (MB), and resveratrol (RSV) on the cognitive functions of 15-month-old mice and their relationship to the maintenance of mitochondrial quality control in the brain and the bacterial composition of the gut microbiome. We have shown that studied compounds enhance mitochondrial biogenesis, mitophagy, and antioxidant defense in the hippocampus of 15-month-old mice via Nrf2/ARE pathway activation, which reduces the degree of oxidative damage to mtDNA. It is manifested in the improvement of short-term and long-term memory. We have also shown that memory improvement correlates with levels of *Roseburia, Oscillibacter, Christensenellaceae* *R-7*, *Negativibacillus,* and *Faecalibaculum* genera of bacteria. At the same time, long-term treatment by MB induced a decrease in gut microbiome diversity, but the other markers of dysbiosis were not observed. Thus, Nrf2/ARE activators have an impact on mitochondrial quality control and are associated with a positive change in the composition of the gut microbiome, which together lead to an improvement in memory in aged mice.

## 1. Introduction

Aging is one of the most serious factors for central nervous dysfunctions such as mild cognitive impairment, dementia, and various neurodegenerative diseases [1]. Most patients with dementia frequently encounter various problems in their daily lives. Even mild cognitive impairment causes trouble to embarrass both the patients and their families [2]. New high effective drugs are required to slow the development of cognitive dysfunction. These drugs should be directed not at symptomatic treatment, but at eliminating the cause of cognitive dysfunctions, which are based on biochemical and molecular genetic causes. At the same time, drug discovery is a time-consuming, laborious, costly, and high-risk process. The success of developing a new molecular drug is about 2%, on average [3]. Drug repositioning is a highly effective, low-cost, and riskless strategy for finding new indications for existing drugs [4].

**Figure 1:**
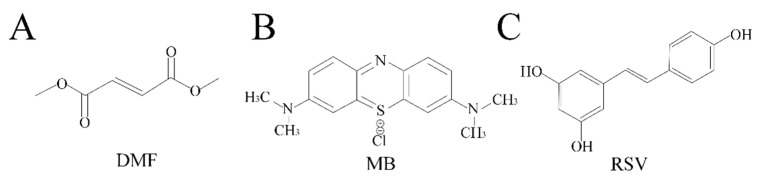
Chemical formula of studied compounds: (**A**) dimethyl fumarate (DMF); (**B**) methylene blue (MB); (**C**) resveratrol (RSV).

Some drugs have the potential to slow down neurodegenerative processes, inhibit the development of dementia, or improve the cognitive phenotype in mild cognitive impairment. In this research, we tested three compounds, which are used as drugs or dietary supplements. Dimethyl fumarate (DMF) is the methyl ester of fumaric acid [5] (Figure 1A). DMF undergoes rapid conversion to its active metabolite, monomethyl fumarate, whose terminal half-life is 1 h [6]. DMF has been approved by the U.S. Food and Drug Administration (FDA) and European Medicines Agency (EMA) as a drug for the treatment of multiple sclerosis (trade name Tecfidera) and for the treatment of moderate-to-severe plaque psoriasis (trade name Skilarence) [7]. Methylene blue (MB), a thiazine dye derivative of phenothiazine, has been described as “the first fully synthetic drug used in medicine” [8] (Figure 1B). Estimated terminal half-life of MB is 5.25 h [9]. Today, MB is the first-line treatment for methemoglobinemia, which is approved by FDA [10]. In addition, MB has been previously used to treat malaria [11]. In recent years, there have been clinical trials of MB for the treatment of Alzheimer’s disease. The first clinical trials [12] were unsuccessful, probably due to incorrect planning of phase III trials and inappropriate use of low doses of MB as a placebo [13]. Today, MB is undergoing clinical trials with other placebo pills [14,15]. Resveratrol (RSV, trans-3,5,4′-trihydroxystilbene) is a polyphenolic phytoalexin that was isolated from different edible parts of plants such as grapes, berries, peanuts, pistachios, and plums (Figure 1C). RSV has received considerable attention for its beneficial effects to health over the past three decades [16]. Unlike the other two drugs, it has not yet been approved by the FDA and is only used as a commercially available supplement. In addition, RSV has a fairly long terminal elimination half-life (8.52 ± 2.27 h) [17]. Clinical trials are currently underway on the effects of RSV supplementation on cognitive function in healthy adults [18].

Common to all three compounds is their ability to activate the factor 2 signaling pathway associated with the NF-E2/antioxidant-responsive element (Nrf2/ARE). Activation of this pathway protects cells from oxidative stress by regulation of the expression of genes that encode proteins with cytoprotective properties, for example, antioxidant enzymes, phase II proteins of xenobiotic detoxification, and anti-inflammatory enzymes as well as metabolic enzymes and regulators involved in maintaining redox homeostasis [19]. Thus, Nrf2/ARE activation may be one of the most promising directions in the treatment of cognitive impairments. Nrf2/ARE activators can provide antioxidant protection and maintain mitochondrial homeostasis associated with the regulation of the number and functionality of mitochondria; they can also suppress degenerative processes in the brain, significantly slowing down the rate of dementia development [20].

However, in addition to mitochondrial dysfunctions, there are several factors that can indirectly influence cognitive changes during aging. In recent years, accumulating data confirm that gut microbiome may modulate host brain function via a microbiome-gut–brain axis. This effect was shown for mild cognitive impairments [21], Alzheimer’s disease [22], Parkinson’s disease [23], depression [24], and schizophrenia [25]. The effect of RSV on the composition of the gut microbiome is well understood [26]. DMF and MB are not well understood in comparison with RSV. There are few studies of the effect of MB on the mouse gut microbiome [27] and DMF effect on the gut microbiota profile of patients with multiple sclerosis [28].

The goal of this study was to comprehensively study the effect of Nrf2/ARE activators on the cognitive abilities of middle-aged mice and their relationship with mitochondrial brain function including mitochondrial biogenesis, mitophagy, antioxidant defense, and mtDNA integrity in parallel with the study of gut microbiome composition in a context of microbiome–gut–brain axis.

The aim of this work was to study the effect of Nrf2/ARE activators on the cognitive abilities of middle-aged mice and their relationship with mitochondrial brain function including mitochondrial biogenesis, mitophagy, antioxidant defense, mtDNA integrity as well as to study gut microbiome composition in the context of the microbiome–gut–brain axis.

## 2. Results

### 2.1. Physiological Tests

None of the studied compounds exhibited a strong effect on behavioral parameters to indicate a change in stressful or exploratory behavior. However, there were changes in the grooming time (F (3, 26) = 5.6928, *p* < 0.01). Post-hoc test showed that RSV increased grooming time compared with the control group (Table 1). In addition, RSV reduced the time that mice spent in the open compartment of the dark–light box (F (3, 27) = 81.163, *p* < 0.001; post-hoc test showed differences between the control and RSV group, *p* < 0.001). DMF decreased the number of transitions between compartments of the dark–light box (F (3, 27) = 5.4463, *p* < 0.01; post-hoc test showed differences between the control and DMF group, *p* < 0.05) (Table 1). There were no differences between groups in the elevated plus-maze and string test (Table 1).

### 2.2. Memory

**Figure 2:**
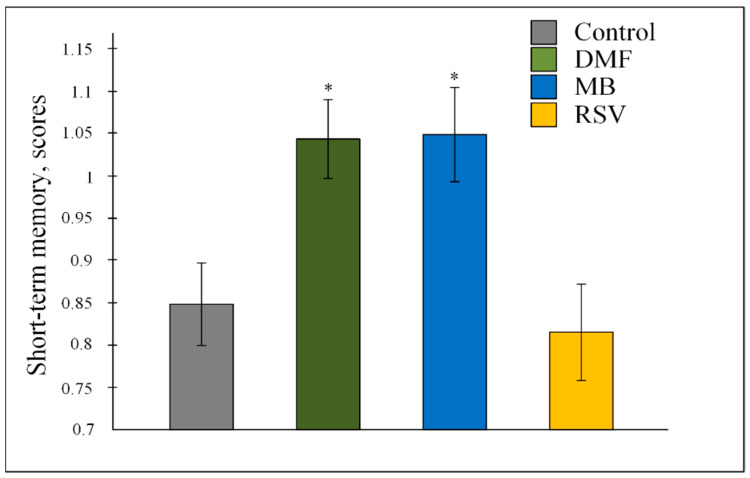
Number of scores in the Water Marris Maze for the assessment of short-term memory. The results expressed as means ± SEM. Control n = 8, DMF = 9, MB = 6, RSV = 7. * *p* < 0.05, comparison of control group and treated groups using Tukey’s post-hoc test.

Nrf2/ARE activators changed the values of short-term memory F (3, 647) = 5.6069, *p* < 0.001. The post-hoc test showed that MB and DMF increased the number of scores by about 20% (both *p* < 0.05) compared with the control (Figure 2).

**Figure 3:**
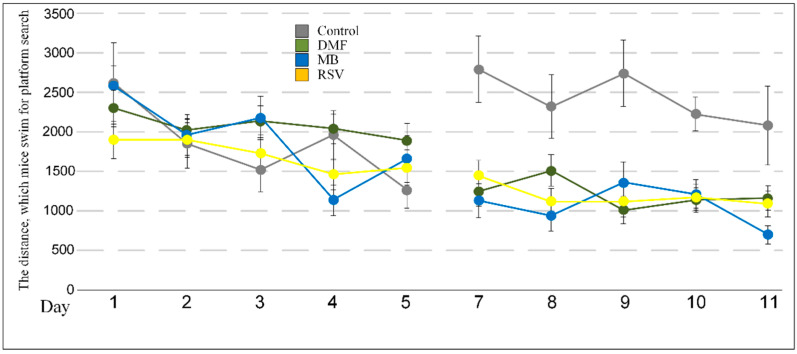
The distance that mice swam for the platform search during acquisition and reversal learning. The results expressed as means ± SEM. Control *n* = 8, DMF = 9, MB = 6, RSV = 8.

The studied compounds did not affect the distance and time that mice spent for the platform search and the time spent in the quadrant with the platform on the days of the study (6th and 12th days) (Table 2, Table 3 and Table 4). Differences were observed during learning. During the acquisition phase of the test, there were differences in the distance that mice swam for the platform search on the 4th day (F (3, 27) = 3.7849, *p* < 0.05). The post-hoc test showed differences between the MB and DMF group (*p* < 0.05) and there were no differences versus the control group. In contrast, during the reversal phase of the test, all compounds tested caused a decrease in the distance that mice spent searching for the platform. On the 7th day, F (3, 27) = 8.1537, *p* < 0.001; on the 8th day, F (3, 27) = 5.3290, *p* < 0.01; on the 9th day F (3, 27) = 8.2114, *p* < 0.001; on the 10th day F (3, 27) = 5.8029, *p* < 0.01; and on the 11th day F (3, 27) = 3.1380, *p* < 0.05 (Table 4, Figure 3).

### 2.3. Gene Expression

**Figure 4:**
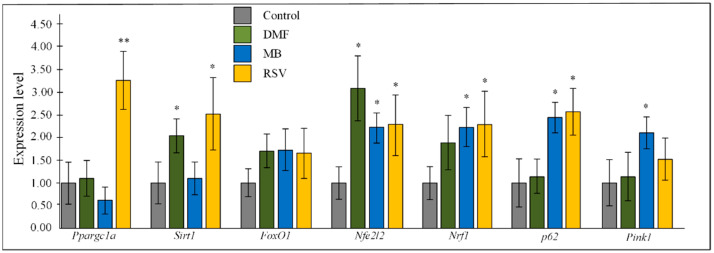
Expression of transcriptional factors in the hippocampus. The results expressed as means ± SEM. Control *n* = 8, DMF = 9, MB = 6, RSV = 7. * *p* < 0.05, ** *p* < 0.01, comparison of control group and treated groups using Tukey’s post-hoc test.

All three studied compounds stimulated a 2–3-fold increase in the expression of the nuclear factor erythroid 2-related factor 2 (*Nfe2l2)* gene in the hippocampus of 15-month-old mice (all *p* < 0.05). DMF increased expression of sirtuin 1 (*Sirt1)* (*p* < 0.05). MB increased expression of mitophagy-related genes *p62* and PTEN-induced kinase 1 (*Pink1*) (both *p* < 0.05) and nuclear respiratory factor 1 (*Nrf1*) (*p* < 0.05). RSV increased expression of PPARG coactivator 1 alpha (*Ppargc1a)* (*p* < 0.01), *Sirt1, Nrf1,* and *p62* (all *p* < 0.05). All three studied Nrf2/ARE activators stimulated more than 50% increase in the expression of the fork box O1 (*FoxO1)* protein compared to the control, but there were no statistically significant changes (Figure 4).

**Figure 5:**
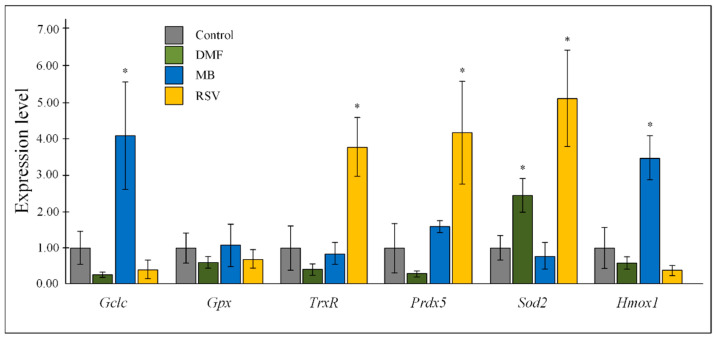
Expression of antioxidants in the hippocampus. The results expressed as means ± SEM. Control *n* = 8, DMF = 9, MB = 6, RSV = 7. * *p* < 0.05, comparison of the control group and treated groups using Tukey’s post-hoc test.

Nrf2/ARE activators affected the expression of antioxidant genes in the hippocampus of 15-month-old mice. DMF induced more than a 2-fold increase in superoxide dismutase 2 (*Sod2)* expression (*p* < 0.05). MB stimulated expression of catalytic subunit of glutamate cysteine ligase (*Gclc)* and heme oxygenase 1 (*Hmox1)*. RSV increased expression of thioredoxin reductase (*TrxR)*, peroxiredoxin-5 (*Prdx5)*, and *Sod2* gene. The effect of the tested compounds on the expression of glutathione peroxidase (Gpx) were not shown (Figure 5).

### 2.4. Copy Number of mtDNA

**Figure 6:**
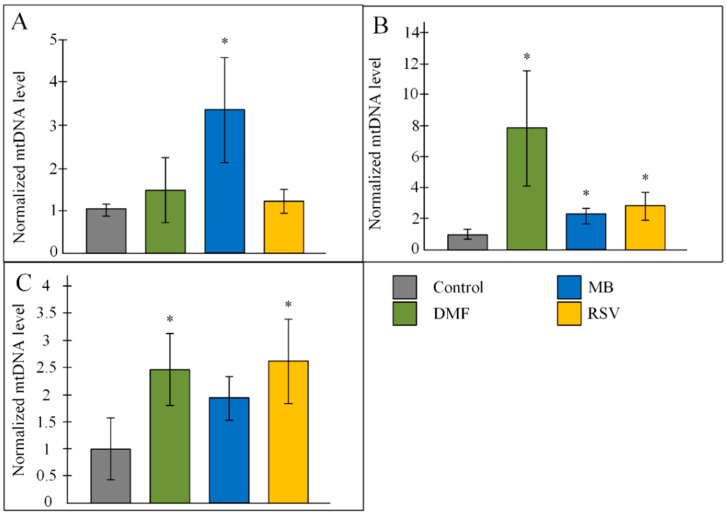
Copy number of mtDNA in the (**A**) hippocampus, (**B**) cortex, (**C**) mid-brain. The results expressed as means ± SEM. Control *n* = 8, DMF = 9, MB = 6, RSV = 7. * *p* < 0.05, comparison of the control group and treated groups using Tukey’s post-hoc test.

MB induced aa 3-fold increase in mtDNA copy number in the hippocampus (*p* < 0.05). DMF and RSV did not affect the copy number mtDNA in the hippocampus (Figure 6A). All three studied Nrf2/ARE activators increased the copy number of mtDNA in the cortex. It should be noted that DMF increased the number of mtDNA copies in the cerebral cortex by eight times compared to the control (*p* < 0.05) (Figure 6B). Mice that received DMF and RSV had a 2.5-fold increased number of mtDNA in the mid-brain (both *p* < 0.05). MB also stimulated an increase in the number of copies of mtDNA, but the changes were not statistically significant (Figure 6C).

### 2.5. mtDNA Damage

**Figure 7:**
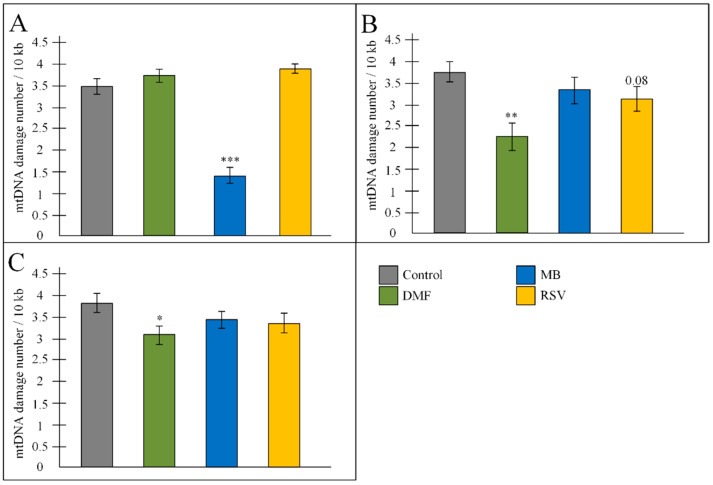
Amount of mtDNA damage in the (**A**) hippocampus, (**B**) cortex, (**C**) mid-brain. The results expressed as means ± SEM. Control n = 8, DMF = 9, MB = 6, RSV = 7. The results expressed as means ± SEM. Control n = 8, DMF = 9, MB = 6, RSV = 8. * *p* < 0.05, ** *p* < 0.01, *** *p* < 0.001, comparison of the control group and treated groups using Tukey’s post-hoc test.

MB more than 2-fold reduced the amount of mtDNA damage in the hippocampus of 15-month-old mice (*p* < 0.001) (Figure 7A). A decrease in the number of damage was observed in fragments 2, 4, 5, 6 (Table 5), which corresponded to the 16s-ND1 region and ND5-ND6-CytB-D-loop region, while RSV in the first and second fragments (12s-16s-ND1 region), in contrast, stimulated an increase in the number of lesions, and DMF caused damage in fragment 3 (ND1-ND2 region) (Table 5).

DMF decreased the number of mtDNA damage in the cortex (*p* < 0.01) due to the reduced damages in the 2nd, 3rd, and 6th fragments (12s-16s-ND1 and D-loop regions) (Figure 7B, Table 5). MB and RSV decreased the amount of mtDNA damage in the D-loop fragment only. For this reason, there was no statistically significant decrease in the average number of damage in the cortex of mice that received MB and RSV (Table 5).

DMF only also decreased the number of mtDNA damage in the midbrain, mainly due to reducing the number of damage in the 2nd fragment. Treatment with MB and RSV also led to a decrease in the amount of mtDNA damage in the 2nd fragment, but these compounds stimulated damage in the D-loop region (Figure 7C, Table 5).

### 2.6. Bacterial Composition of Gut Microbiome

**Figure 8:**
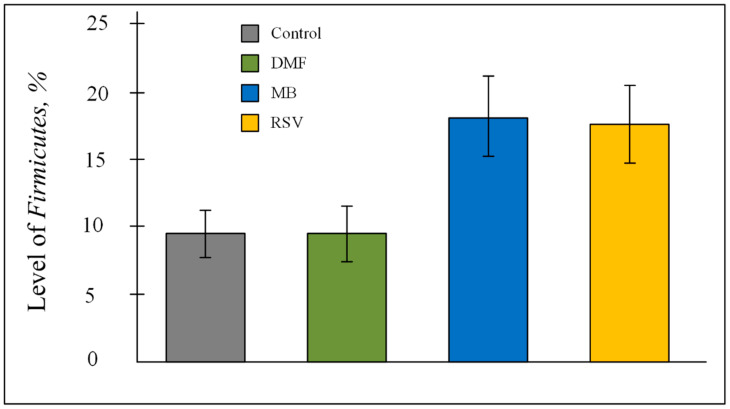
Level of Firmicutes phylum level in the gut microbiome. The results expressed as means ± SEM. Control *n* = 8, DMF = 8, MB = 6, RSV = 6.

PCR analysis showed that more than 95% of the bacteria in the gut microbiome were *Bacteroidetes* and *Firmicutes* (Table 6). ANOVA showed that Nrf2/ARE activators affect the percentage of bacteria of the *Firmicutes* phylum (F (3, 24) = 4.2200, *p* < 0.05). However, the post-hoc test did not show differences between the control and treated groups. A tendency to an increase in *Firmicutes* level was shown for the MB group (*p* = 0.069) and RSV group (*p* = 0.07) (Figure 8).

There was a negative correlation between the level of *Verrucomicrobia* and distance (r_s_ = −0.494) and time (r_s_ = −0.465) that mice spent to search the goal platform on 12th day. At the same time, *Verrucomicrobia* negatively correlated with the time that mice spent to search the goal platform on 12th day (r_s_ = −0.398). *Tenericutes* level positively correlated with distance (r_s_ = 0.474) and time (r_s_ = −0.436) that mice spent to search the goal platform on the 6th day. Time spent in the quadrant with the platform positively correlated with *Deferribacteres* level (r_s_ = 0.407) and “*Candidatus* Saccharibacteria” (r_s_ = 0.387) (Table S1). Multivariate correlation analysis showed a negative correlation between scores of short-term memories and *Epsilonproteobacteria* level (R = −0.504) (Table S3).

NGS analysis showed that genus *Prevotella* (*Bacteroidetes* phylum) was dominant in the control group (0.135 ± 0.031), DMF group (0.135 ± 0.058), and RSV group (0.160 ± 0.027). In the MB group, genus *Lachnospiraceae* UCG-001 (*Firmicutes* phylum) was dominant (0.147 ± 0.062), while the *Prevotella* amount was lower (0.096 ± 0.018) (Table 7).

There was a negative correlation between the level of *Mycoplasma* and time on the 12th day (r_s_ = −0.453), between time in the goal quadrant on the 12th day (r_s_ = −0.451) and on the 6th day (r_s_ = 0.506). In addition, we observed a positive correlation between the level of *Mycoplasma* and number of scores of short-term memory (r_s_ = 0.456). Level of *Roseburia* negatively correlated with distance (r_s_ = −0.493) and time (r_s_ = −0.425) that mice spent to search the goal platform on the 12th day. Level of *Oscillibacter* negatively correlated with distance (r_s_ = −0.407) and time (r_s_ = −0.474) that mice spent to search the goal platform on the 12th day. Level of *Christensenellaceae* R-7 group bacteria negatively correlated with distance (r_s_ = −0.421) and time (r_s_ = −0.412) that mice spent to search the goal platform on the 12th day. There was a negative correlation between level of *Muribaculum* and time on the 6th day (r_s_ = −0.470), between time in the goal quadrant on the 6th day (r_s_ = −0.400) and distance on the 6th day (r_s_ = −0.464). Level of *Bilophila* negatively correlated with distance (r_s_ = −0.549) and time in the goal quadrant (r_s_ = −0.562) that mice spent to search the goal platform on the 12th day. There was a negative correlation between the level of *Negativibacillus* and distance on the 6th day (r_s_ = −0.453). There was a positive correlation between time in a goal quadrant on 6th day and level of *Faecalibaculum* (r_s_ = 0.415) (Table S2).

In our research, a number of bacterial genera was associated with memory impairment. We observed a negative correlation between time in a goal quadrant on the 6th day and level of *Streptococcus* (r_s_ = −0.397) and level of *Odoribacter* (r_s_ = −0.434). Level of *Acinetobacter* was correlated with distance (r_s_ = 0.462) and time (r_s_ = 0.471) that mice spent searching the goal platform on the 6th day as well as *Clostridium* (r_s_ = 0.442 with distance and r_s_ = 0.467 with time). There was a positive correlation between time that mice spent to search the goal platform on the 12th day and level of *Dubosiella* (r_s_ = 0.422) and level of *Desulfovibrio* (r_s_ = 0.403). Time that mice spent searching the goal platform on the 6th day correlated with *Prevotellaceae* UCG-001 (r_s_ = 0.397). The distance that mice swam for the platform search correlated with *Tyzzerella* level (r_s_ = 0.413) and UCG-002 (Family *Oscillospiraceae*) level (r_s_ = 0.419) (Table S2). Multivariate correlation analysis showed strong positive correlation between *Bifidobacterium* and time that mice spent in goal quadrant at day 12 (R = 0.795). Other average and weak correlations are represented in Table S4.

## 3. Discussion

**Figure 9:**
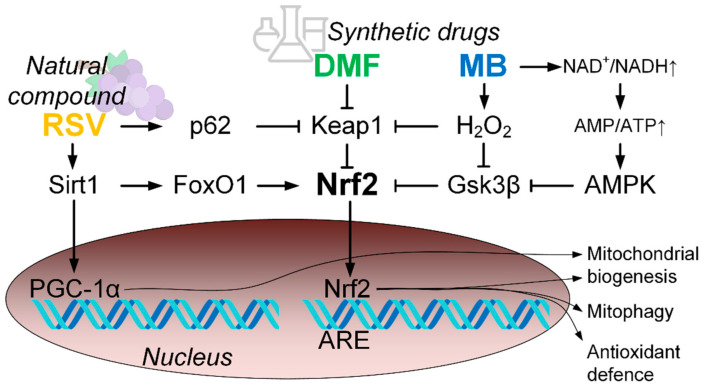
Scheme of the activation of the Nrf2/ARE signal pathway by dimethyl fumarate (DMF); methylene blue (MB); resveratrol (RSV).

Analysis of the expression data shows that DMF, MB, and RSV activate the Nrf2/ARE signal pathway in the hippocampus of 15-month-old mice. All three studied compounds doubled the expression of the *Nfe2l2* gene, which encodes the Nrf2 protein (Figure 4). *Nfe2l2* contains ARE-like sequences in the promoter region and Nrf2 may activate its own gene expression, leading to increased production of Nrf2 protein [29]. It is well known that DMF, MB, and RSV activate the Nrf2/ARE signal pathway (Figure 9) [30,31,32]. DMF covalently interacts with the reactive cysteines lying in the BTB and IVR regions of Kelch-like ECH-associated protein 1 (Keap1). In addition, DMF binds to the Nrf2-binding site (bottom region of Keap1-DC), and to the top region of Keap1-DC, near blade II [30]. RSV forms H-bonds with Ser49, Asn100, Arg101, Leu196, and Ser288 of the Kelch domain and Pi–cation interaction with Arg101 (motifs in zebrafish) [31]. RSV also activates Nrf2 through stimulation of the Sirt1/FoxO1 pathways [32]. These data have been confirmed by qPCR. We showed that RSV increases the expression of the *Sirt1* gene and there is a tendency to increase the expression of *FoxO1*, but the changes are not statistically significant (Figure 4). In addition, RVS activation of Nrf2 may be due to p62-dependent autophagic degradation of Keap1 [33] (Figure 9). We showed that RSV induced an increase in p62 expression (Figure 4).

There are currently no data showing that MB directly activates Nrf2 due to interaction with Keap1 or glycogen synthase kinase 3 beta (GSK3β). However, there are several indirect mechanisms that can lead to the activation of Nrf2. It is known that MB can accept electrons from NADH and perform alternative electron transport in the electron–transport chain [34]. This leads to a shift in the NAD^+^/NADH equilibrium toward NAD^+^. This leads to the activation of AMP-activated protein kinase (AMPK), which can activate the Nrf2/ARE signaling pathway [35] (Figure 9). In addition, MB in the electron transfer process can increase the H_2_O_2_ production rate [36,37]. We have previously shown that this process is not associated with a significant increase in oxidative stress in the brain, but can cause compensatory effects [37,38]. H_2_O_2_ can activate Nrf2 by disrupting its interaction with both Keap1 and GSK3β (Figure 9).

There are various consequences of the activation of the Nrf2/ARE signaling pathway. It is known that this signaling pathway plays an important role in the regulation of antioxidant defense [39], which is manifested in the fact that Nrf2 activators to varying degrees activated the expression of antioxidant genes (Figure 5). This is actually the target for the drug because there is an age-dependent decrease in antioxidant mechanisms [40]. It should be noted that not all Nrf2 activators increase the expression of all studied antioxidants, most of all increased by RSV treatment (4-fold increase in *TrxR* and *Prdx5* expression and 5-fold increase in *Sod2* expression) (Figure 5). This is not surprising because RSV is a phenolic stilbenoid compound. Generally, phenolic compounds are powerful antioxidants [41].

Regulation of mitochondrial pool volume in the cell is an important part of the maintenance of mitochondrial quality control [42]. For a long time, it was believed that “PGC-1α is a master regulator of mitochondrial biogenesis” [43]. In recent years, evidence has begun to accumulate that this is not clear and that there are other ways of mitochondrial biogenesis regulation, one of which is the Nrf2/ARE signaling pathway [44]. We found that one of the Nrf2/ARE activators (RSV) could activate the Sirt1/PGC-1α axis due to the increase in expression of both *Sirt1* and *Ppargc1a* (PGC-1α encoded gene) (Figure 9). In addition, we observed that RSV increased *Nrf1* expression (Figure 4), which plays a critical role in the transcriptional regulation of mtDNA transcription process [45]. At the same time, the level of mtDNA in the hippocampus was not increased in mice treated with RSV. Hippocampal mtDNA increased in MB-treated mice (Figure 6a). Similarly, we found a MB-induced increase in *Nrf1* expression. However, no increase in the expression of genes involved in the activation of the Sirt1/PGC-1α axis was found (Figure 4). These data confirm that PGC-1α is not major regulator of mitochondrial biogenesis and other pathways can regulate the level of mitochondria in the cell [44]. It should be noted that in the forebrain, all three studied compounds increased the number of mtDNA copies, while in the mid-brain, the increase in the number of mtDNA copies was caused by DMF and RSV (Figure 6).

Mitophagy is another important process that regulates the number of mitochondria in a cell. [46]. MB and RSV stimulate expression of both *p62* and *Pink1* genes (Figure 4), which may indicate that mitophagy processes are activated. Mitophagy plays an important role in maintaining the integrity of mitochondria and various mitochondrial components [46]. We found that MB stimulated a decrease in the amount of mtDNA damage in the hippocampus (Figure 7, Table 5). It is likely that this may be due to the activation of the mitophagy process. Surprisingly, RSV did not induce a decrease in the amount of mtDNA damage (Figure 7, Table 5), although it also activated the expression of genes associated with mitophagy and stimulated antioxidant defense more strongly than other Nrf2 activators (Figure 5). Obviously, there are many other factors that can affect the integrity of mtDNA and other mitochondrial components.

We showed that Nrf2/ARE activators, in general, improve some parameters of the memory of 15-month-old mice where DMF and MB increased score numbers in short-term memory (Figure 2). We did not observe a direct improvement in the values of long-term memory, which may be induced by Nrf2/ARE activators. We showed that mice treated with DMF, MB, and RSV swam less distance before finding the platform compared with the control group of mice during reversal learning (Table 2, Figure 3). Reversal learning in the water Morris maze reveals whether or not animals can extinguish their initial learning of the platform’s position and acquire a direct path to the new goal position [47]. Thus, DMF, MB, and RSV allow for a more rapid switch away from the old goal location to the new goal compared with the control. However, by the 6th day after the change in platform position, the control mice showed the same result as the treated mice (Table 2, Figure 3).

Early on, it has been shown that RSV treatment significantly improved the learning and memory in rats with vascular dementia [48], rats with H-89-induced deficits on spatial memory [49], rats with bilateral common carotid artery occlusion [50], juvenile mice with high-calorie diet-induced memory dysfunction [51], and mice with AlCl_3_ and D-galactose-induced cognitive deficits [52]. Additionally, RSV improved learning, memory, and mood function among 25-month-old mice [53]. Nevertheless, a meta-analysis of 225 patients showed that RSV had no significant impact on factors related to memory and cognitive performance including learning ability, delayed recall, retention, and recognition [54]. DMF treatment alleviated long-term memory deficits induced by lipopolysaccharide [55] and prevented the disruption of spatial reference and working memory induced by streptozotocin [56]. The neuroprotective effect of MB began to be studied in the late 1970s when it was shown that MB acts as an inhibitory avoidance response [57]. Furthermore, it has been shown that MB administration during the memory consolidation period restored the memory retention impaired by the inhibition of cytochrome oxidase [58]. MB was associated with a 7% increase in correct responses during memory retrieval [59].

We hypothesize that the cognitive improvements that the studied compounds stimulate are due to a number of factors. We have studied several aspects of mitochondrial quality control in the brain. However, cognitive functions are closely related to other, at first glance, non-obvious factors. Recently, the gut–brain axis has been actively discussed. Communication between the gut microbiome and the cognitive function can occur via neuronal, immunological, and endocrine pathways. For example, it is well known that the microbiota regulates the hypothalamus–pituitary–adrenal axis [60]. PCR analysis showed that Nrf2/ARE activators impact the *Firmicutes* level (Table 6, Figure 8). Previously, it has been shown that the *Firmicutes* level was significantly correlated with higher scores on the cognitive test on neurologically-healthy older adult people [61].

Spearman’s rank correlation analysis showed a positive correlation between *Verrucomicrobia* level and cognitive function, and a negative correlation between *Tenericutes* level and cognitive function (Table S1). A positive correlation between *Verrucomicrobia* level and cognitive functions was noted for the neurologically-healthy older adult people [61]. Increases in *Verrucomicrobia* level suppress neurodegeneration [62]. *Verrucomicrobia* reverses cognitive dysfunction including impaired spatial working memory and recognition of new objects, restores brain metabolism [63], and alleviates memory impairment caused by high fat in mice [64]. Patients with cognitive impairment had a lower abundance of *Tenericutes* [65]. This is at odds with our data that showed a positive correlation between *Tenericutes* level and distance (r_s_ = 0.474) and time (r_s_ = 0.436) spent on the platform search in the water Morris maze (Table S1). Multivariate correlation analysis showed a negative correlation between scores of short-term memories and *Epsilonproteobacteria* level (R= −0.504) (Table S3). Not all bacteria in this phillum are pathogenic, but the increase in their number suggests that there may be damage to organs or organ systems, which leads to memory impairment [66].

We showed positive correlation between memory values and level of *Roseburia, Oscillibacter, Christensenellaceae* R-7, *Negativibacillus*, and *Faecalibaculum* genera (Table S2). It has earlier been shown that cognitive decline coincided with a decrease in *Roseburia* [66] and opposite memory improvement was associated with a significant increase in the abundance of *Roseburia* [67]. Improved cognitive function of APP/PS1 mice was associated with increase in *Oscillibacter* level [68]. Level of *Faecalibaculum* was also positively associated with cognitive function [68,69,70,71]. The role of *Christensenellaceae* R-7 and *Negativibacillus* in cognition has not been previously considered (Table S2).

We showed positive correlation between memory values and level of Streptococcus, Odoribacter, Acinetobacter, Clostridium, Dubosiella, Desulfovibrio, Prevotellaceae UCG-001, Tyzzerella, and UCG-002 level (Family Oscillospiraceae). Odoribacter level increased significantly in APP/PS1 [72] and aged [73] mice with impaired spatial learning. Desulfovibrio was also associated with memory decline, induced by a high-fat diet [74,75]. At the same time, some of our data are at odds with the previously obtained results. Increased Streptococcus was associated with probiotic-induced prevention of diet-induced memory deficits [76], while our data showed negative correlation with time that mice spent in a goal quadrant (Table S2). Additionally, decrease in Prevotellaceae was associated with memory deficits [77], while our data showed positive correlation with time that mice spent for the platform search (Table S2).

Some bacteria genera (*Mycoplasma, Bilophila, Muribaculum*) showed contradictory effects in the various values of memory search (Suppl. 2). Early, positive correlation between cognition and bacteria level were shown for *Bilophila* [78] and *Muribaculum* [71,79]. Previous studies have found no link between cognitive performance and *Mycoplasma* levels [80,81,82], which is partly confirmed by our contradictory data. It should be kept in mind that we have not shown a direct effect of drugs on the content of these bacteria genera (Table 7), therefore, we cannot say that the addition of Nrf2/ARE activators has a strong effect on the bacterial composition of the intestinal microbiota.

**Figure 10:**
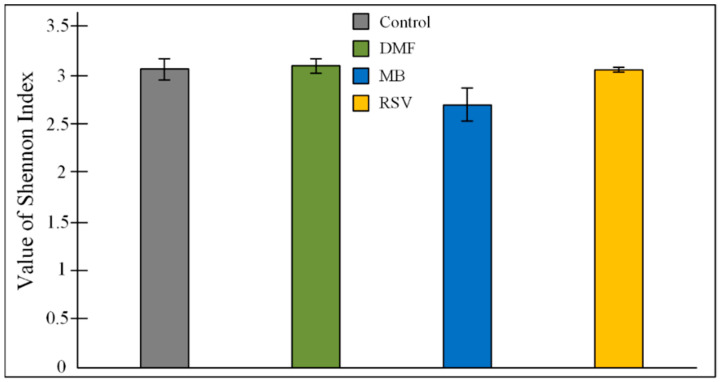
Value of Shannon index (*H*) of alfa-diversity. The results are expressed as means ± SEM. Control n = 8, DMF = 6, MB = 6, RSV = 6.

**Figure 11:**
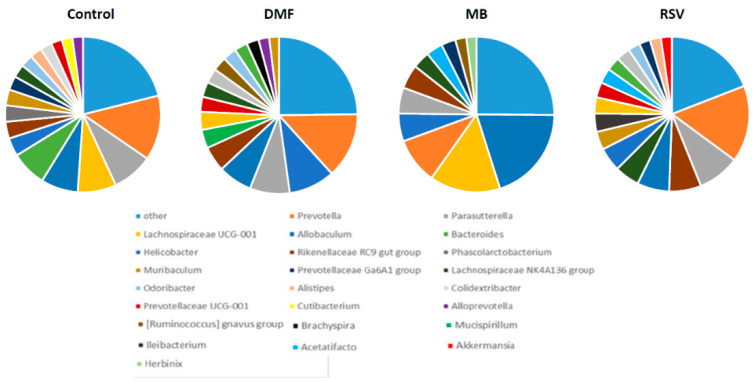
Predominant genera in the gut microbiome. Control n = 8, DMF = 6, MB = 6, RSV = 6.

It is known that even non-antibiotic drugs have a notable impact on the overall architecture of the intestinal microbiome [83]. This implies the long-term treatment by drugs that are aimed at slowing down the processes of cognitive impairment, which can increase the degree of the effect of the drug on the intestinal composition of the microbiome. Recently, we found that treatment by a high concentration of MB (50 mg/kg/day) during one month induced dysbiosis, which manifested in an increase in *Proteobacteria* level [27]. Classically thought to be markers of dysbiosis in inflammatory bowel disease are increase in the *Proteobacteria* and *Bacteroidetes* phyla bacteria, decrease in the number of *Firmicutes* phylum bacteria, and decrease in the alpha-diversity index [84]. In this research (MB concentration 15 mg/kg/day for 3 months), we showed no changes in the *Proteobacteria* and *Bacteroidetes* level, and a tendency to increase in *Firmicutes* level (Table 6, Figure 8). However, MB-treated mice had a tendency decrease in alfa-diversity. Shannon index for MB group was 2.699 ± 0.169, while for the control group it was 3.063 ± 0.108; for the DMF group it was 3.100 ± 0. 069; for RSV it was 3.067 ± 0.023 (Figure 10). The ANOVA test showed that F (3, 22) = 2.9073, *p* = 0.0574. The post-hoc test did not show differences between the control and MB-treated groups, but there was a tendency toward a decrease in Shannon index (*p* = 0.0832). It is believed that normal diversity is when Shannon index ≥3.0 [85]. In the other groups of mice (control group and DMF, RSV groups), the Shannon index was more than 3.0 (Figure 10). MB mediated decrease of bacterial diversity in the gut microbiome also manifested in the decreasing number of predominant genera. Gut microbiome of MB-treated mice contained 11 genera with OTU of more than 0.02, while the control mice and RSV-treated mice contained 17 genera and DMF-treated mice contained 16 genera (Figure 11). Thus, these results and our previous data [27] show some evidence of MB-induced dysbiosis, but we cannot say that MB causes dysbiosis, since the other markers of dysbiosis are not observed. It is known that dysbiosis altered brain function and induced depressive-like behavior [86]. We also did not find that MB could increase stress levels or suppress exploratory behavior in mice (Table 1), though the PROVEPHARM SAS company, which manufactures ProVayBlue (MB-based FDA-approved drug for acquired methemoglobinemia) warns that MB can cause a confusional state and warns against driving or operating machines (Drugs FDA: FDA-Approved Drugs; New Drug Application (NDA): 204630) [87].

In contrast, not one marker of dysbiosis was shown for the DMF and RSV treated groups (Table 6 and Table 7). Previously, the pilot study did not detect a major effect of DMF on the gut microbiota composition [28]. RSV, in contrast, had a prebiotic effect on bacteria, which could have a positive effect on body weight and fat mass [88,89].

Thus, we can conclude that DMF, MB, and RSV can activate Nrf2/ARE signaling, which leads to an enhancement of mitochondrial biogenesis, mitophagy, and antioxidant defense in the hippocampus of aged mice. This is manifested in the improvement of short-term and long-term memory during normal aging-associated cognitive deterioration. We cannot directly extrapolate these results to cognitive dysfunction and dementia, which can be caused by neurodegenerative diseases such as Alzheimer’s disease, Parkinson’s disease, learning behavior disorder, progressive supranuclear palsy, frontotemporal dementia and others, which have specific pathogenetic mechanisms that differ from normal aging. These results expand our understanding of the effect of Nrf2/ARE activators on mitochondrial quality control, its relationship with the change in the composition of the gut microbiome, and improvement in memory in health aged mice.

## 4. Materials and Methods

### 4.1. Animals

Fifteen-month-old mice (C57Bl/6 strain) were used in the experiment. Mice were obtained from Stolbovaya Nursery (Moscow region, Russia) and kept under standard conditions of vivarium at t = 25 °C, relative humidity of at least 40%, and 12-h light/dark cycle. Standard laboratory diet (Ssniff Spezialdiaten GmbH, Soest, Germany) and drinking water were available ad libitum. Mice were divided into four groups: (1) control group (mice received pure water, n = 8); (2) DMF group (mice received 10 mg/kg/day DMF dissolved in water, n = 9); (3) MB group (mice received 15 mg/kg/day MB dissolved in water, n = 6); and (4) RSV group (mice received 20 mg/kg/day RSV dissolved in water, n = 8). All chemicals were obtained from Sigma-Aldrich (St. Louis, MO, USA). All experiments with animals were performed in accordance with the guidelines of the Voronezh State University Ethical Committee on Biomedical Research (Animal Care and Use Section, protocol N42-01a dated 16 March 2020).

Mice received the compound for 50 days. On the 50th day of the experiment, an open field test was carried out. On the 52nd day of the experiment, a dark–light box test was carried out. On the 54th day of the experiment, an elevated-plus-maze test was carried out. On the 56th day of the experiment, a string test was carried out. Water Morris test for assessing long-term memory was performed between days 57 and 68 days. Morris water-maze test for assessing short-term memory was performed between days 69 and 89 days. On the 90th day of the experiment, mice feces were collected for microbiome analysis. Further mice were sacrificed at the age of 18 months.

Mice were sacrificed by a mixture (1 mL/kg) of xylazine (10 mg/kg) and ketamine (90 mg/kg) administered intraperitoneally. Afterward, mice were decapitated and brains were dissected. In this research, we used hippocampus compartment for biochemical and expression analysis. A MtDNA study was conducted on the hippocampus, forebrain, and ventral mid-brain.

### 4.2. Physiological Tests

For assessment of anxiety-related and exploratory behavior, we used an open field test. The mice were placed in the corner of the open arena (60 × 60 × 40 cm) with five randomly distributed holes (0.5 cm diameter) for 5 min. We evaluated locomotor activity of mice (s), time spent in the center (s), number of exits to the center, number of hole-pocking acts, the number of rearing acts and defecation acts, the duration (s), and the number of grooming acts.

For assessment of anxiety-related behavior, we used a dark–light box. A box contained two compartments. The light compartment (24 × 20 × 25 cm) took up 2/3 of the device and did not have a lid, while the dark compartment (12 × 20 × 25 cm) took up 1/3 and was covered with a lid. The compartments were connected by a door (4 × 5 cm). The animal was placed in the light compartment and had the ability to move freely between the compartments for 5 min. We evaluated the time spent in the light or dark part of the chamber, and the number of transitions between compartments.

For exploratory behavior assessment, the elevated plus-maze test was used. The maze contained two open arms (30 × 5 cm) and two enclosed arms (30 × 5 × 15 cm). Each mouse was placed on the crossing arms and the 5 min test session was initiated. We evaluated the time spent in the open arms.

For assessment of the strength and endurance of mice, we used the string test. The mice, holding their forelimbs, were suspended on a string 50 cm long at a height of 50 cm above the surface. A cushion underneath was also provided to prevent injury to falling mice. Next, the mice were rated according to the following criteria: 1—hang by two forepaws; 2—attempt to climb onto the string; 3—two forepaws + one or both hind paws; and 4—four paws + tail around the string. If the mice fell, they received from 0 to 0.9 points (depending on a time of falling). For example, a mouse received 0.25 points if it fell in 15 s of the trial or 0.5 points if it fell in 30 s of trial. If a mouse reached the end of the string, it received from 5 to 5.9 points (depending on a time of escape). For example, a mouse received 5.75 points if it escaped in 15 s of the trial or 5.5 points if it escaped in 30 s of the trial. For each mouse, there were two trials with 30 min between trials.

### 4.3. Morris Water Maze

To study the short-term and long-term memory of the mice, the Morris water maze was used. The experiment was performed in a round pool (154 cm diameter) with a round platform (15 cm diameter) in the target quadrant. The pool was filled with colored water so that the platform was hidden below the water surface (0.5 cm). The study of long-term memory took place in two stages: learning and probe. During learning, the animals were trained for five days and four trials, and each animal had to remember the location of the platform. On day 6 (probe), each animal was given one trial to reach the platform under 1 min. The second stage (reversal) of the study of reference memory includes the same stages as the first, with the only difference being that the platform should be located in the opposite quadrant of the arena. On day 12, each animal was given one trial to reach the platform under 1 min. To study the long-term memory, the following parameters were measured: distance from the starting point to platform (cm); time that mice spent for the platform search (s); and the time the animal spent in the quadrant with the platform (s).

To study short-term memory, the animal was given only two attempts with an interval of 15 s in order to find the platform. The study lasted 21 days. The difference in time (s) to reach the platform is an important indicator. The level of short-term memory was expressed in scores, which were calculated according to Equation (1) when latency of the second trial was more than the latency of the first trial and according to Equation (2) when the latency of the second trial was less than the latency of the first. When a mouse did not find the platform, it received 0 scores.

(1) $$Score=1-\left(\frac{2nd\;trial*\frac{100}{1st\;trial}}{100}-1\right)$$

(2) $$Score=1+\left(\frac{2nd\;trial*\frac{100}{1st\;trial}}{100}\right)$$

### 4.4. DNA and RNA Isolation

Total DNA from the brain compartment was isolated using the Proba-GS Kit (DNA-Technology, Moscow, Russia) according to the protocol. Isolation of total RNA was performed using the ExtractRNA Kit (Evrogen, Moscow, Russia) according to the protocol. Qualitative analysis of DNA and RNA was carried out using electrophoresis in 2% agarose gel in 1× TAE-buffer.

### 4.5. Measurement of mtDNA Copy Number

The amount of mtDNA copy number was estimated by quantitative PCR of the mtDNA fragment using the Bio-Rad CFX96^TM^ Real-Time System and 1× qPCR mix-HS SYBR kit (Evrogen, Russia). The mtDNA primer sequences were as follows:

F: 5′-ACGAGGGTCCAACTGTCTCTTA-3′;

R: 5′-AGCTCCATAGGGTCTTCTCGT-3′.

The *Gapdh* and *18*s rRNA genes of nuclear DNA were used as a reference. The primer sequences were as follows:

*Gapdh*—F: 5′-GGCTCCCTAGGCCCCTCCTG-3′;

R: 5′-TCCCAACTCGGCCCCCAACA-3′;

*18s*—F: 5′-CGGCTACCACATCCAAGGAA-3′;

R: 5′-GCTGGAATTACTGTGGCT-3′.

qPCR cycling conditions were: initial denaturation at 95 °C for 3 min followed by 35 cycles: denaturation 95 °C for 10 s, primer annealing at 59 °C for 30 s, and elongation at 72 °C for 30 s.

Normalized mtDNA level relative to nuclear DNA was calculated using standard Equation (3):

(3) $$mtDNA\;level=2^{\left(-\Delta \Delta Cq\right)}$$

### 4.6. mtDNA Damage Measurement

The amount of mtDNA damage was estimated by quantitative long-range PCR. Each PCR reaction contained 1× of Encyclo polymerase, 1× Encyclo buffer, 0.2 mM of each dNTP (all Evrogen, Russia), 1× SYBR GreenMasterMix (BioDye, Moscow, Russia), and a mix of forward and reverse primers in a total volume of 20 μL. qPCR cycling conditions were: initial denaturation at 95 °C for 3 min; 35 cycles: denaturation 95 °C for 30 s, primer annealing at 59 °C for 30 s, and elongation at 72 °C for 270 s.

The primers were designed previously [90]. The amount of mtDNA damage was calculated per 10,000 bp according to Equation (4).

(4) $$mtDNA\;damage=1-(2^{-\left(\Delta long-\Delta short\;\right)})*10000/fragment\;lenght$$

where Δ *long* = *Cq* control − *Cq* experiment for the long fragment and Δ *short* = *Cq* control − cq experiment for the short fragment.

### 4.7. Gene Expression Analysis

RNA was used to obtain cDNA using MMLV reverse transcriptase (Evrogen, Russia) on an Eppendorf Mastercycler personal thermal cycler (Eppendorf, Hamburg, Germany). qPCR cycling conditions were: initial denaturation at 95 °C for 3 min followed by 35 cycles: denaturation 95 °C for 10 s, primer annealing at 59 °C for 30 s, and elongation at 72 °C for 30 s. The primer sequences are presented in Table 8. For calculation of normalized expression, standard Bio-Rad CFX Manager software was used.

### 4.8. Analysis of Gut Microbiome Using PCR

Bacteria in the mice feces were analyzed according to Yang et al. (2015) by quantitative PCR of the mtDNA fragment using a Bio-Rad CFX96^TM^ Real-Time System and 1× qPCR mix-HS SYBR Kit (Evrogen, Russia). The content of bacteria of a particular phylum was determined using the Equation (5).

(5) $$\%\;bacteria=\frac{E_{Univ}^{{Cq}_{Univ}}}{E_{Spec}^{{Cq}_{Spec}}}\;\times \;100\%$$

where E_Univ_ is PCR efficiency with the universal primers; E_Spec_ is PCR efficiency with the phylum-specific primers; Cq_Univ_ is the number of quantitation cycle with the universal primers; and Cq_Spec_ is the number of quantitation cycle with the phylum-specific primers

### 4.9. Analysis of Gut Microbiome Using NGS

We selected the variable region V3 of the *16s* rRNA gene to study the microbiome using sequencing on the Ion Torrent PGM. Bacterial DNA was amplified with the universal direct 337F forward primer and reverse 518R primer. The primer sequences were as follows:

337F: 5′-GACTCCTACGGGAGGCWGCAG-3′;

518R:5′-GTATTACCGCGGCTGCTGG-3′.

PCR was performed using a 5X ScreenMix-HS Master Mix (Evrogen, Russia) in the following regime: 94 °C for 4 min followed by 37 cycles of 94 °C for 30 s, 53 °C for 30 s, and 72 °C for 30 s with the final elongation at 72 °C for 5 min.

PCR products were purified with AMPure XP magnetic beads (Beckman Coulter, Brea, CA, USA) and used for constructing sequencing libraries using NEBNext Fast DNA Library Prep (New England Biolabs, Ipswich, MA, USA) as recommended by the manufacturer. Barcoding was performed using Ion Xpress barcode adapters (Thermo Fisher Scientific, Waltham, MA, USA). Library DNA concentration was determined by qPCR using Library Quantification Kit Ion Torrent Platforms (Kapa Biosystems, Wilmington, MA, USA).

Sequencing was performed on the Ion Torrent PGM system using Ion PGM Hi-Q View Sequencing Kit, Ion OneTouch 2System, and Ion PGM Hi-Q View OT2 Kit (Thermo Fisher Scientific, Wilmington, MA, USA). Libraries were sequenced using an Ion Chip 318.

### 4.10. Statistical Analysis

Statistical analysis was performed using Statistica 10 (StatSoft. Inc., Tulsa, OK, USA). The results were expressed as means ± SEM. The results of the physiological tests and bacterial composition of gut microbiome were analyzed by one-way analysis of variance (ANOVA). Tukey’s post-hoc test was used to determine the significance level. Correlation analysis was performed using Spearman’s rank correlation (r_s_). For calculation of normalized expression and copy number of mtDNA, standard Bio-Rad CFX Manager software was used.

Sequencing results were obtained as binary alignment map (BAM) files that were converted into the FASTQ format using the SAMtools v.1.2 software. The reads were filtered according to the reading quality based on the expected number of errors using the maximum expected error cutoff of 1.0 [91]. The samples were pooled and unique sequences were identified before searching for the operational taxonomic units (OTUs). We searched for the OTUs using the UNOISE2 algorithm, which reduces the noise through error correction [92,93]. We combined all reads for all samples to generate OTUs and compile an OTU table.

Filtration of reads, identification of unique sequences, and clusterization in order to search for the OTUs were performed using either USEARCH v.10.0.240 or VSEARCH v.2.8.2 software. Microbial genus in the samples were identified using the SILVA database v.123 (https://www.arb-silva.de (accessed on 29 April 2021)).

Microbiota diversity was quantified using Shannon index (*H*) according Equation (6).

(6) $$H=-\overset{S}{\underset{i=1}{\sum }}pi(\ln pi)$$

where *p_i_* is often the proportion of bacteria genus belonging to the *i*th species in the microbiome.

## Figures and Tables

**Table 1:** Results of physiological tests: open field, dark-light box, elevated plus-maze (EPM), string test.

Test	Open Field					
Indicator	Horizontal Activity (s)	Time in the Center (s)	Entering in the Center (Number)	Rearing (Number)	Hole-Poking (Number)	Grooming (s)
Control	114.62 ± 14.49	19.87 ± 7.13	5.87 ± 1.78	15.25 ± 2.25	4.5 ± 0.91	10.62 ± 1.44
DMF	124.00 ± 7.41	16.00 ± 4.07	5.12 ± 0.67	12.75 ± 1.23	3.12 ± 0.69	9.25 ± 2.61
MB	83.67 ± 14.27	10.5 ± 2.66	4.83 ± 1.51	14.00 ± 2.67	2.66 ± 0.80	9.00 ± 3.38
RSV	90.75 ± 12.89	10.66 ± 3.38	4.5 ± 1.18	7.62 ± 2.09	5.5 ± 2.38	24.25 ± 4.38*
**Test**	**Open Field**		**Dark-Light Box**		**EPM**	**String**
**Indicator**	**Grooming** **(Number)**	**Defecation**	**Time in the Open Compartment (s)**	**Transition between Compartments (Number)**	**Time in the Open Arm (s)**	**Scores** **(Number)**
Control	1.75 ± 0.16	1.62 ± 0.86	235.25 ± 8.64	15.75 ± 2.68	4.37 ± 1.61	2.8 ± 0.33
DMF	4.12 ± 2.42	0.25 ± 0.25	253.22 ± 9.16	7.67 ± 1.43 *	8.44 ± 2.53	1.82 ± 0.39
MB	1.5 ± 0.22	1.5 ± 0.5	231.83 ± 7.50	16.83 ± 1.42	6.83 ± 2.09	3.5 ± 0.43
RSV	2.75 ± 0.65	2.5 ± 1.44	64.37 ± 12.64 ***	8.37 ± 2.14	5.75 ± 4.34	3.2 ± 0.40

The results expressed as means ± SEM. Control *n* = 8, DMF = 9, MB = 6, RSV = 8. * *p* < 0.05, *** *p* < 0.001, comparison of the control group and treated groups using Tukey’s post-hoc test.

**Table 2:** The distance that mice swam for the platform search.

Acquisition	Learning					Probe
Start SW	Day 1	Day 2	Day 3	Day 4	Day 5	Day 6
	N; E; SE; NW	SE; N; NW; E	NW; SE; E; N	E; NW; N; SE	N; SE; E; NW	NW
Control	2611 ± 514	1852 ± 306	1511 ± 266	1956 ± 313	1265 ± 232	1401 ± 410
DMF	2512 ± 230	2018 ± 151	2130 ± 201	2035 ± 187	1887 ± 222	2897 ± 359
MB	2573 ± 266	1960 ± 257	2174 ± 276	1134 ± 192	1654 ± 295	1682 ± 464
RSV	1896 ± 238	1894 ± 220	1724 ± 236	1461 ± 195	1544 ± 223	1376 ± 385
**Reversal**	**Learning**					**Probe**
Start NE	Day 7	Day 8	Day 9	Day 10	Day 11	Day 12
	S; W; NW; SE	NW; S; SE; W	SE; NW; W; S	W; SE; S; NW	S; NW; W; SE	SE
Control	2774 ± 423	2306 ± 398	2726 ± 420	2212 ± 213	2064 ± 498	1324 ± 443
DMF	1228 ± 184 **	1491 ± 195	996 ± 174 ***	1122 ± 153 **	1142 ± 154 *	1062 ± 401
MB	1113 ± 217 **	920 ± 188 **	1335 ± 266*	1195 ± 179 *	682 ± 116 ***	1838 ± 334
RSV	1430 ± 195 *	1107 ± 165 *	1229 ± 201 **	1160 ± 163 **	1076 ± 168 *	1691 ± 338

The results expressed as means ± SEM. Control *n* = 8, DMF = 9, MB = 6, RSV = 8. * *p* < 0.05, ** *p* < 0.01, *** *p* < 0.001, comparison of the control group and treated groups using Tukey’s post-hoc test.

**Table 3:** The time that mice spent on the platform search.

Acquisition	Learning					Probe
Start SW	Day 1	Day 2	Day 3	Day 4	Day 5	Day 6
	N; E; SE; NW	SE; N; NW; E	NW; SE; E; N	E; NW; N; SE	N; SE; E; NW	NW
Control	38.3 ± 6.2	28.4 ± 5.4	35.5 ± 10.0	29.5 ± 4.5	34.5 ± 6.5	24.5 ± 8.5
DMF	47.3 ± 3.3	49.9 ± 2.6	46.7 ± 3.4	44.2 ± 3.3	36.9 ± 3.8	49.2 ± 5.6
MB	45.9 ± 4.0	34.3 ± 4.6	37.3 ± 4.5	24.3 ± 4.1	30.7 ± 5.6	29.3 ± 9.7
RSV	41.2 ± 4.3	37.6 ± 3.8	31.6 ± 4.3	29.2 ± 3.9	24.5 ± 3.6	29.4 ± 8.9
**Reversal**	**Learning**					**Probe**
Start NE	Day 7	Day 8	Day 9	Day 10	Day 11	Day 12
	S; W; NW; SE	NW; S; SE; W	SE; NW; W; S	W; SE; S; NW	S; NW; W; SE	SE
Control	19.7 ± 3.6	17.6 ± 3.5	20.9 ± 3.3	21.5 ± 2.6	19.8 ± 5.5	22.1 ± 7.4
DMF	21.7 ± 3.3	31.7 ± 3.9	17.4 ± 3.2	21.6 ± 3.4	23.0 ± 3.5	16.8 ± 6.6
MB	17.7 ± 3.1	15.1 ± 3.0	19.1 ± 3.8	18.4 ± 2.8	13.1 ± 2.5	31.0 ± 3.9
RSV	26 ± 3.8	22.4 ± 3.6	26.1 ± 4.3	23.1 ± 3.8	25.9 ± 3.7	33.0 ± 8.6

The results expressed as means ± SEM.

**Table 4:** The time that mice spent in the quadrant with the platform.

Acquisition	Learning					Probe
Start SW	Day 1	Day 2	Day 3	Day 4	Day 5	Day 6
	N; E; SE; NW	SE; N; NW; E	NW; SE; E; N	E; NW; N; SE	N; SE; E; NW	NW
Control	10.3 ± 1.62	9.3 ± 1.38	11.4 ± 2.95	7.5 ± 0.98	5.9 ± 0.87	8.5 ± 3.09
DMF	11 ± 0.99	17.4 ± 1.52	10.9 ± 0.98	14.2 ± 1.55	10.8 ± 1.30	10.6 ± 1.83
MB	11.8 ± 1.72	13.9 ± 2.29	12.2 ± 1.42	13.9 ± 2.68	10.1 ± 1.81	7.8 ± 2.40
RSV	9.7 ± 0.97	12.7 ± 1.66	10.9 ± 1.61	9.5 ± 1.47	8.3 ± 1.27	10.9 ± 3.79
**Reversal**	**Learning**					**Probe**
Start NE	Day 7	Day 8	Day 9	Day 10	Day 11	Day 12
	S; W; NW; SE	NW; S; SE; W	SE; NW; W; S	W; SE; S; NW	S; NW; W; SE	SE
Control	5.7 ± 0.90	5.2 ± 0.43	7.2 ± 0.94	6.5 ± 0.51	8.3 ± 1.23	4.6 ± 1.05
DMF	7.9 ± 1.14	7.6 ± 0.95	5.7 ± 0.79	4.9 ± 0.79	6.0 ± 0.80	5.6 ± 1.83
MB	6.3 ± 1.08	5.3 ± 0.86	5.3 ± 0.76	5.8 ± 0.84	3.9 ± 0.56	10.7 ± 2.16
RSV	7.6 ± 1.18	6.4 ± 1.19	5.2 ± 1.01	5.4 ± 1.15	5.1 ± 0.73	9.5 ± 3.37

The results expressed as means ± SEM.

**Table 5:** Amount of mtDNA damage.

**Hippocampus**				
	Control	DMF	MB	RSV
1 fragment	2.05 ± 0.30	2.97 ± 0.30	1.20 ± 0.34	3.68 ± 0.19 ***
2 fragment	2.33 ± 0.24	3.83 ± 0.71	0.80 ± 0.34 **	4.63 ± 0.26 ***
3 fragment	2.19 ± 0.54	3.46 ± 0.21 *	1.50 ± 0.48	3.29 ± 0.28
4 fragment	3.84 ± 0.28	4.03 ± 0.20	1.72 ± 0.61 **	3.82 ± 0.27
5 fragment	3.84 ± 0.23	4.09 ± 0.18	1.39 ± 0.32 ***	4.22 ± 0.24
6 fragment	4.75 ± 0.30	5.17 ± 0.17	1.82 ± 0.59 ***	4.39 ± 0.21
**Forebrain**				
1 fragment	2.26 ± 0.35	1.86 ± 0.47	3.16 ± 0.35	3.03 ± 0.42
2 fragment	5.35 ± 0.34	3.60 ± 0.79 *	6.23 ± 0.24	5.51 ± 0.59
3 fragment	4.74 ± 0.14	2.94 ± 0.51 ***	4.72 ± 0.16	4.32 ± 0.44
4 fragment	1.71 ± 0.47	0.47 ± 0.69	1.26 ± 0.53	1.23 ± 0.48
5 fragment	2.50 ± 0.74	0.97 ± 0.67	3.00 ± 0.62	3.17 ± 0.53
6 fragment	5.92 ± 0.45	3.59 ± 1.05 *	1.55 ± 1.24 **	1.47 ± 0.98 ***
**Mid-Brain**				
1 fragment	2.30 ± 0.37	2.90 ± 0.29	3.35 ± 0.35 *	3.16 ± 0.46
2 fragment	7.54 ± 0	2.27 ± 0.72 ***	2.48 ± 0.88 ***	2.43 ± 0.88 ***
3 fragment	4.15 ± 0.45	2.88 ± 0.55	2.98 ± 0.59	2.62 ± 0.52 *
4 fragment	2.71 ± 0.38	3.45 ± 0.29	3.14 ± 0.17	3.39 ± 0.24
5 fragment	3.1 ± 0.53	2.71 ± 0.57	3.60 ± 0.35	2.74 ± 0.45
6 fragment	3.1 ± 0.65	4.28 ± 0.59	5.05 ± 0.45 *	5.84 ± 0.48 **

The results expressed as means ± SEM. Control *n* = 8, DMF = 9, MB = 6, RSV = 7. * *p* < 0.05, ** *p* < 0.01, *** *p* < 0.001, comparison of the control group and treated groups using Tukey’s post-hoc test.

**Table 6:** Phyla of bacteria in gut microbiome. Results of PCR analysis.

	Control	DMF	MB	RSV
*Bacteroidetes*	85.20 ± 2.74	84.19 ± 2.31	75.44 ± 4.31	79.70 ± 3.01
*Firmicutes*	9.45 ± 1.76	9.48 ± 2.07	18.18 ± 2.97	17.60 ± 2.86
*Actinobacteria*	0.26 ± 0.04	0.25 ± 0.08	0.26 ± 0.08	0.11 ± 0.03
*Betaproteobacteria*	2.24 ± 1.05	1.20 ± 0.36	0.90 ± 0.24	0.75 ± 0.17
*Epsilonproteobacteria*	0.46 ± 0.29	0.30 ± 0.07	0.20 ± 0.07	0.60 ± 0.21
*Delta*- and *Gammaproteobacteria*	1.26 ± 0.30	2.24 ± 0.59	2.78 ± 0.69	2.05 ± 0.90
*«Candidatus* Saccharibacteria*»*	0.42 ± 0.12	0.80 ± 0.29	0.55 ± 0.15	0.47 ± 0.11
*Deferribacteres*	0.29 ± 0.11	0.69 ± 0.36	1.60 ± 0.88	0.52 ± 0.23
*Tenericutes*	0.00 ± 0.00	0.02 ± 0.01	0.02 ± 0.01	0.00 ± 0.00
*Verrucomicrobia*	0.41 ± 0.15	0.82 ± 0.67	0.06 ± 0.01	0.17 ± 0.04

**Table 7:** Gut microbiome (NGS).

Phylum	Class	Family	Genus	Control	DMF	MB	RSV
*Abditibacteriota*	*Abditibacteriaceae*	*Abditibacteriaceae*	*Abditibacterium*	0.001 ± 0.000	0.000 ± 0.000	0.000 ± 0.000	0.000 ± 0.000
*Actinobacteriota*	*Actinobacteria*	*Propionibacteriaceae*	*Cutibacterium*	0.022 ± 0.022	0.001 ± 0.000	0.001 ± 0.000	0.001 ± 0.000
		*Bifidobacteriaceae*	*Bifidobacterium*	0.005 ± 0.001	0.004 ± 0.003	0.003 ± 0.001	0.013 ± 0.007
		*Corynebacteriaceae*	*Corynebacterium*	0.002 ± 0.000	0.000 ± 0.000	0.001 ± 0.000	0.001 ± 0.000
	*Coriobacteriia*	*Atopobiaceae*	*Olsenella*	0.000 ± 0.000	0.000 ± 0.000	0.001 ± 0.000	0.001 ± 0.000
*Proteobacteria*	*Alphaproteobacteria*	*Rhizobiales*	*Methylobacterium-Methylorubrum*	0.005 ± 0.005	0.000 ± 0.000	0.000 ± 0.000	0.000 ± 0.000
		*Caulobacterales*	*Asticcacaulis*	0.002 ± 0.000	0.000 ± 0.000	0.000 ± 0.000	0.000 ± 0.000
	*Gammaproteobacteria*	*Burkholderiales*	*Parasutterella*	0.085 ± 0.025	0.082 ± 0.055	0.054 ± 0.013	0.088 ± 0.021
			*Burkholderia-Caballeronia-Paraburkholderia*	0.006 ± 0.000	0.000 ± 0.000	0.000 ± 0.000	0.000 ± 0.000
		*Enterobacteriaceae*	*Escherichia-Shigella*	0.002 ± 0.002	0.000 ± 0.000	0.000 ± 0.000	0.001 ± 0.000
		*Xanthomonadaceae*	*Pseudoxanthomonas*	0.004 ± 0.000	0.000 ± 0.000	0.000 ± 0.000	0.001 ± 0.000
		*Moraxellaceae*	*Acinetobacter*	0.001 ± 0.000	0.000 ± 0.000	0.000 ± 0.000	0.001 ± 0.000
		*Rhodanobacteraceae*	*Rudaea*	0.001 ± 0.000	0.000 ± 0.000	0.000 ± 0.000	0.000 ± 0.000
*Bacteroidota*	*Bacteroidia*	*Prevotellaceae*	*Prevotella*	0.135 ± 0.031	0.135 ± 0.058	0.096 ± 0.018	0.160 ± 0.027
			*Prevotellaceae UCG-001*	0.023 ± 0.005	0.031 ± 0.014	0.014 ± 0.004	0.032 ± 0.008
			*Prevotellaceae Ga6A1 group*	0.029 ± 0.008	0.015 ± 0.009	0.030 ± 0.011	0.023 ± 0.010
			*Alloprevotella*	0.022 ± 0.007	0.022 ± 0.021	0.010 ± 0.002	0.012 ± 0.003
		*Marinifilaceae*	*Odoribacter*	0.026 ± 0.006	0.028 ± 0.013	0.014 ± 0.004	0.025 ± 0.004
		*Bacteroidaceae*	*Bacteroides*	0.074 ± 0.025	0.027 ± 0.009	0.018 ± 0.003	0.029 ± 0.004
		*Muribaculaceae*	*Muribaculum*	0.033 ± 0.006	0.020 ± 0.010	0.017 ± 0.004	0.038 ± 0.013
		*Tannerellaceae*	*Parabacteroides*	0.008 ± 0.002	0.005 ± 0.003	0.004 ± 0.001	0.005 ± 0.001
		*Rikenellaceae*	*Rikenella*	0.006 ± 0.001	0.006 ± 0.004	0.007 ± 0.001	0.006 ± 0.001
			*Alistipes*	0.025 ± 0.005	0.019 ± 0.011	0.011 ± 0.003	0.023 ± 0.006
			*Rikenellaceae RC9 gut group*	0.035 ± 0.004	0.052 ± 0.029	0.050 ± 0.027	0.067 ± 0.025
*Campilobacterota*	*Campylobacteria*	*Helicobacteraceae*	*Helicobacter*	0.038 ± 0.007	0.095 ± 0.070	0.058 ± 0.021	0.050 ± 0.007
*Firmicutes*	*Clostridia*	*Lachnospiraceae*	*Lachnospiraceae UCG-001*	0.080 ± 0.025	0.037 ± 0.017	0.147 ± 0.062	0.034 ± 0.014
			*Herbinix*	0.014 ± 0.004	0.012 ± 0.016	0.021 ± 0.015	0.007 ± 0.001
			*[Ruminococcus] gnavus group*	0.013 ± 0.002	0.029 ± 0.022	0.023 ± 0.008	0.016 ± 0.005
			*Acetatifactor*	0.006 ± 0.001	0.010 ± 0.014	0.034 ± 0.019	0.031 ± 0.012
			*Stomatobaculum*	0.002 ± 0.001	0.004 ± 0.002	0.003 ± 0.001	0.008 ±0.003
			*Lachnospiraceae NK4A136 group*	0.027 ± 0.005	0.031 ± 0.020	0.036 ± 0.006	0.052 ± 0.011
			*Tuzzerella*	0.004 ± 0.001	0.006 ± 0.006	0.002 ± 0.000	0.002 ± 0.001
			*Tyzzerella*	0.003 ± 0.001	0.003 ± 0.000	0.001 ± 0.000	0.001 ± 0.000
			*Roseburia*	0.008 ± 0.006	0.003 ± 0.002	0.003 ± 0.001	0.007 ± 0.004
			*GCA-900066575*	0.003 ± 0.001	0.002 ± 0.001	0.001 ± 0.000	0.002 ± 0.001
			*ASF356*	0.002 ± 0.000	0.002 ± 0.002	0.002 ± 0.000	0.001 ± 0.000
			*Blautia*	0.001 ± 0.000	0.002 ± 0.002	0.001 ± 0.000	0.001 ± 0.001
			*[Eubacterium] hallii group*	0.001 ± 0.000	0.002 ± 0.001	0.001 ± 0.000	0.001 ± 0.000
			*A2*	0.002 ± 0.000	0.002 ± 0.001	0.004 ± 0.001	0.002 ± 0.001
			*Lachnospiraceae UCG-003*	0.001 ± 0.000	0.001 ± 0.000	0.003 ± 0.001	0.001 ± 0.000
			*[Eubacterium] xylanophilum group*	0.003 ± 0.002	0.003 ± 0.002	0.002 ± 0.000	0.003 ± 0.001
			*[Eubacterium] fissicatena group*	0.002 ± 0.000	0.002 ± 0.001	0.003 ± 0.001	0.002 ± 0.000
			*Mobilitalea*	0.001 ± 0.000	0.003 ± 0.001	0.001 ± 0.000	0.001 ± 0.000
			*Lachnoanaerobaculum*	0.004 ± 0.001	0.007 ± 0.006	0.005 ± 0.002	0.007 ± 0.001
		*Oscillospiraceae*	*Oscillibacter*	0.014 ± 0.003	0.018 ± 0.013	0.007 ± 0.001	0.011 ± 0.001
			*Colidextribacter*	0.024 ± 0.005	0.030 ± 0.012	0.016 ± 0.001	0.029 ± 0.002
			*UCG-003*	0.004 ± 0.001	0.009 ± 0.005	0.003 ± 0.001	0.007 ± 0.001
			*Flavonifractor*	0.003 ± 0.001	0.003 ± 0.002	0.002 ± 0.000	0.003 ± 0.001
			*UCG-002*	0.002 ± 0.001	0.002 ± 0.001	0.000 ± 0.000	0.001 ± 0.000
			*Intestinimonas*	0.009 ± 0.002	0.010 ± 0.007	0.006 ± 0.001	0.009 ± 0.002
		*Ruminococcaceae*	*Ruminococcus*	0.018 ± 0.004	0.019 ± 0.015	0.013 ± 0.004	0.011 ± 0.005
			*Incertae Sedis*	0.000 ± 0.001	0.003 ± 0.002	0.004 ± 0.001	0.003 ± 0.002
			*Negativibacillus*	0.004 ± 0.001	0.002 ± 0.001	0.002 ± 0.000	0.003 ± 0.001
			*Angelakisella*	0.002 ± 0.001	0.003 ± 0.001	0.001 ± 0.000	0.001 ± 0.000
			*Paludicola*	0.002 ± 0.000	0.001 ± 0.000	0.001 ± 0.000	0.001 ± 0.000
			*Anaerotruncus*	0.004 ± 0.001	0.004 ± 0.002	0.004 ± 0.001	0.001 ± 0.000
			*[Eubacterium] siraeum group*	0.003 ± 0.001	0.002 ± 0.001	0.002 ± 0.001	0.008 ± 0.004
		*Clostridiaceae*	*Clostridium sensu stricto 1*	0.001 ± 0.000	0.002 ± 0.001	0.003 ± 0.001	0.001 ± 0.000
		*Anaerovoracaceae*	*[Eubacterium] nodatum group*	0.002 ± 0.001	0.002 ± 0.001	0.001 ± 0.000	0.001 ± 0.000
			*Family XIII UCG-001*	0.001 ± 0.000	0.003 ± 0.001	0.001 ± 0.000	0.001 ± 0.000
		*Monoglobaceae*	*Monoglobus*	0.001 ± 0.000	0.001 ± 0.000	0.003 ± 0.002	0.002 ± 0.002
		*Christensenellaceae*	*Christensenellaceae R-7 group*	0.001 ± 0.000	0.001 ± 0.001	0.001 ± 0.000	0.001 ± 0.000
		*Peptococcaceae*	*Peptococcus*	0.000 ± 0.000	0.000 ± 0.000	0.001 ± 0.001	0.000 ± 0.000
	*Bacilli*	*Erysipelotrichaceae*	*Allobaculum*	0.077 ± 0.020	0.070 ± 0.069	0.200 ± 0.079	0.067 ± 0.017
			*Ileibacterium*	0.017 ± 0.006	0.019 ± 0.030	0.011 ± 0.004	0.038 ± 0.016
			*Dubosiella*	0.003 ± 0.002	0.004 ± 0.002	0.004 ± 0.002	0.007 ± 0.002
			*Faecalibaculum*	0.001 ± 0.000	0.004 ± 0.003	0.001 ± 0.000	0.002 ± 0.001
		*Lactobacillaceae*	*Lactobacillus*	0.012 ± 0.005	0.008 ± 0.002	0.015 ± 0.003	0.008 ± 0.003
			*Atopostipes*	0.001 ± 0.000	0.001 ± 0.000	0.001 ± 0.000	0.001 ± 0.000
		*Streptococcaceae*	*Streptococcus*	0.004 ± 0.003	0.001 ± 0.000	0.001 ± 0.000	0.003 ± 0.001
		*Acholeplasmataceae*	*Anaeroplasma*	0.001 ± 0.000	0.002 ± 0.003	0.003 ± 0.003	0.001 ± 0.000
		*Staphylococcaceae*	*Staphylococcus*	0.002 ± 0.002	0.000 ± 0.000	0.000± 0.000	0.000 ± 0.000
			*Jeotgalicoccus*	0.001 ± 0.000	0.000 ± 0.000	0.003 ± 0.002	0.001 ± 0.000
		*Exiguobacteraceae*	*Exiguobacterium*	0.004 ± 0.000	0.000 ± 0.000	0.000 ± 0.000	0.000 ± 0.000
		*Erysipelatoclostridiaceae*	*Candidatus Stoquefichus*	0.001 ± 0.000	0.001 ± 0.000	0.001 ± 0.000	0.004 ± 0.003
			*Erysipelatoclostridium*	0.001 ± 0.000	0.000 ± 0.000	0.000 ± 0.000	0.001 ± 0.000
		*Streptococcaceae*	*Lactococcus*	0.001 ± 0.001	0.000 ± 0.000	0.000 ± 0.000	0.002 ± 0.001
		*Mycoplasmataceae*	*Ureaplasma*	0.000 ± 0.000	0.002 ± 0.002	0.001 ± 0.000	0.002 ± 0.001
			*Mycoplasma*	0.002 ± 0.000	0.001± 0.000	0.000 ± 0.000	0.001 ± 0.000
	*Negativicutes*	*Acidaminococcaceae*	*Phascolarctobacterium*	0.034 ± 0.021	0.019 ± 0.035	0.002 ± 0.001	0.001 ± 0.000
		*Sporomusaceae*	*Pelosinus*	0.001 ± 0.000	0.000 ± 0.000	0.000 ± 0.000	0.000 ± 0.000
*Deferribacterota*	*Deferribacteres*	*Deferribacteraceae*	*Mucispirillum*	0.010 ± 0.003	0.038 ± 0.052	0.014 ± 0.011	0.006 ± 0.001
*Desulfobacterota*	*Desulfovibrionia*	*Desulfovibrionaceae*	*Desulfovibrio*	0.001 ± 0.000	0.002 ± 0.002	0.001 ± 0.001	0.001 ± 0.000
			*Bilophila*	0.001 ± 0.000	0.002 ± 0.001	0.001 ± 0.000	0.001 ± 0.000
*Spirochaetota*	*Leptospirae*	*Leptospiraceae*	*Leptospira*	0.002 ± 0.000	0.000 ± 0.000	0.000 ± 0.000	0.000 ± 0.000
	*Brachyspirae*	*Brachyspiraceae*	*Brachyspira*	0.004 ± 0.001	0.025 ± 0.040	0.004 ± 0.003	0.002 ± 0.001
*Patescibacteria*	*Saccharimonadia*	*Saccharimonadaceae*	*Candidatus Saccharimonas*	0.005 ± 0.001	0.008 ± 0.005	0.003 ± 0.001	0.008 ± 0.001
*Verrucomicrobiota*	*Verrucomicrobiae*	*Akkermansiaceae*	*Akkermansia*	0.012 ± 0.006	0.009 ± 0.013	0.010 ± 0.006	0.023 ± 0.014

**Table 8:** Primer sequences for expression measurement.

Gene	Forward Primer 5′–3′	Reverse Primer 5′–3′
*18s*	CGGCTACCACATCCAAGGAA	GCTGGAATTACTGTGGCT
*Gapdh*	GGCTCCCTAGGCCCCTCCTG	TCCCAACTCGGCCCCCAACA
*Ppargc1a*	ATGTGTCGCCTTCTTGCTCT	CACGACCTGTGTCGAGAAAA
*Sirt1*	CTGTTTCCTGTGGGATACCTGACT	ATCGAACATGGCTTGAGGATCT
*FoxO1*	GGGTCTGTCTCCCTTTCCTC	TCAGTGGCATTCAGCAGGTA
*Nfe2l2*	CTCTCTGAACTCCTGGACGG	GGGTCTCCGTAAATGGAAG
*Nrf1*	AGCACGGAGTGACCCAAA	TGTACGTGGCTACATGGACCT
*P62*	GCCAGAGGAACAGATGGAGT	TCCGATTCTGGCATCTGTAG
*Pink1*	GAGCAGACTCCCAGTTCTCG	GTCCCACTCCACAAGGATGT
*Gclc*	GCAGCTTTGGGTCGCAAGTAG	TGGGTCTCTTCCCAGCTCAGT
*Gpx*	AGTCCACCGTGTATGCCTTCT	GAGACGCGACATTCTCAATGA
*Txnr2*	GATCCGGTGGCCTAGCTTG	TCGGGGAGAAGGTTCCACAT
*Prdx5*	GGCTGTTCTAAGACCCACCTG	GGAGCCGAACCTTGCCTTC
*Sod2*	CAGACCTGCCTTACGACTATGG	CTCGGTGGCGTTGAGATTGTT
*Hmox1*	CACGCATATACCCGCTACCT	CCAGAGTGTTCATTCGAGCA

## Data Availability

Data available as Supplementary Materials.

## Supplementary Materials

The following are available online at https://www.mdpi.com/article/10.3390/ph14070607/s1, Table S1: Correlation between bacterial composition of gut microbiome (PCR results) and values of memory; Table S2: Correlation between bacterial composition of gut microbiome (PCR results) and values of memory; Table S3: Multivariate correlation between bacterial composition of gut microbiome (PCR results) and values of memory; Table S4: Multivariate correlation between bacterial composition of gut microbiome (PCR results) and values of memory; Table S5: Open field results; Table S6: Dark-light box results; Table S7: Elevated plus maze results; Table S8: String test results; Table S9: Long-term memory. Distance; Table S10: Long-term memory. Time; Table S11: Long-term memory. Time in goal quadrant; Table S12: Short-term memory. Time; Table S13: Short-term memory. Scores; Table S14: Microbiome. PCR; Table S15: Microbiome. NGS.
